# γ-Radiation Promotes Immunological Recognition of Cancer Cells through Increased Expression of Cancer-Testis Antigens *In Vitro* and *In Vivo*

**DOI:** 10.1371/journal.pone.0028217

**Published:** 2011-11-29

**Authors:** Anu Sharma, Beata Bode, Roland H. Wenger, Kuno Lehmann, Alessandro A. Sartori, Holger Moch, Alexander Knuth, Lotta von Boehmer, Maries van den Broek

**Affiliations:** 1 Department of Oncology, University Hospital Zurich, Zurich, Switzerland; 2 Department of Pathology, University Hospital Zurich, Zurich, Switzerland; 3 Institute of Physiology, University of Zurich, Zurich, Switzerland; 4 Department of Visceral Surgery, University Hospital Zurich, Zurich, Switzerland; 5 Institute of Molecular Cancer Research, University of Zurich, Zurich, Switzerland; University of Chicago, United States of America

## Abstract

**Background:**

γ-radiation is an effective treatment for cancer. There is evidence that radiotherapy supports tumor-specific immunity. It was described that irradiation induces de novo protein synthesis and enhances antigen presentation, we therefore investigated whether γ-radiation results in increased expression of cancer-testis (CT) antigens and MHC-I, thus allowing efficient immunological control. This is relevant because the expression of CT-antigens and MHC-I on tumor cells is often heterogeneous. We found that the changes induced by γ-radiation promote the immunological recognition of the tumor, which is illustrated by the increased infiltration by lymphocytes after radiotherapy.

**Methods/Findings:**

We compared the expression of CT-antigens and MHC-I in various cancer cell lines and fresh biopsies before and after *in vitro* irradiation (20 Gy). Furthermore, we compared paired biopsies that were taken before and after radiotherapy from sarcoma patients. To investigate whether the changed expression of CT-antigens and MHC-I is specific for γ-radiation or is part of a generalized stress response, we analyzed the effect of hypoxia, hyperthermia and genotoxic stress on the expression of CT-antigens and MHC-I. *In vitro* irradiation of cancer cell lines and of fresh tumor biopsies induced a higher or de novo expression of different CT-antigens and a higher expression of MHC-I in a time- and dose-dependent fashion. Importantly, we show that irradiation of cancer cells enhances their recognition by tumor-specific CD8+ T cells. The analysis of paired biopsies taken from a cohort of sarcoma patients before and after radiotherapy confirmed our findings and, in addition showed that irradiation resulted in higher infiltration by lymphocytes. Other forms of stress did not have an impact on the expression of CT-antigens or MHC-I.

**Conclusions:**

Our findings suggest that γ-radiation promotes the immunological recognition of the tumor. We therefore propose that combining radiotherapy with treatments that support tumor specific immunity may result in increased therapeutic efficacy.

## Introduction

γ-radiation or radiotherapy is one of the most widely used treatments for cancer [1]. Irradiation induces death of tumor cells [2], [3], but there is accumulating evidence that adaptive immunity significantly contributes to the efficacy of radiotherapy [4]. For example, irradiated tumors in patients and in mice are more often infiltrated by leukocytes than the unirradiated tumors [5], [6], [7] and very recent studies in preclinical models showed that the efficacy of radiotherapy depends on the presence of CD8^+^ T cells [8]. The fact that tumors are targeted and controlled by CD8^+^ T cells is suggested by the increased tumor incidence in immunosuppressed patients [9], [10], [11] and by the fact that tumor-specific immunity can be detected in cancer patients [12], [13], [14], [15]. As the recognition of tumor cells by CD8^+^ T cells depends on the presentation of tumor-associated antigens (TAAs) in the context of MHC-I molecules, the often-heterogeneous expression of TAA and/or MHC-I within a tumor negatively impacts on the efficacy of tumor-specific immunity. In the present study we asked the specific question whether irradiation induces or up-regulates the expression of a prominent group of TAAs, the so-called CT-antigens. The CT-antigens form an extended family of antigens that are expressed in a large variety of malignancies but are absent from healthy tissue except for the testis and placenta [16], [17]. Cancer patients often develop spontaneous immune responses towards CT-antigens, which illustrates their immunogenicity [18]. Due to their immunogenicity and restricted pattern of expression, CT-antigens are considered promising targets for immunotherapy in cancer patients [19], [20]. We observed that irradiation induced a higher or a *de novo* expression of different CT-antigens as well as an up-regulation of MHC-I expression in multiple cancer cell lines and in fresh, *ex vivo* irradiated tumor biopsies. Importantly, comparison of paired tumor sections obtained from sarcoma patients before and after irradiation showed up-regulated or *de novo* expression of MHC-I and CT-antigens and the concomitant increase of infiltrating CD8^+^ T cells, suggesting that irradiation mobilizes local, tumor-specific immune responses. Furthermore, our findings indicate that a combination of radiotherapy and active immunization with relevant CT-antigens may be a treatment modality with higher efficacy compared to either therapy alone.

## Materials and Methods

### Ethics Statement

The ethics committee "Ethical committee of the canton of Zurich" specifically approved this study (Study No: EK-1017).

### Cells

#### Cell lines

MDA-MB-469, MDA-MB-231 and MCF 7 (breast cancer cell lines), MCF 10A (normal breast cell line, immortalized, non-transformed), Saos, LM5, 143B, HOS, HU09, and M132 (osteosarcoma cell lines), A549, H460, Calu1 and Calu3 (lung cancer cell lines), SK-MEL-37 (melanoma cell line) and PC3 and DU145 (prostate cancer cell lines) were obtained from American Type Culture Collection (Manassas, VA). The osteosarcoma cell lines were a gift from Dr. Bruno Fuchs (Department of Orthopedics, University Clinic Balgrist, Zurich). All cell lines and biopsies were cultured in RPMI 1640 medium (Invitrogen, Carlsbad, CA) containing 10% fetal bovine serum (FBS; Sigma-Aldrich Corp. St. Louis, MO), L-glutamine and antibiotics. PC3 and DU145 were cultured in DMEM medium (Invitrogen), containing 10% FBS, L-glutamine and antibiotics.

#### Primary human cells

Human foreskin keratinocytes were obtained as a gift from Dr. Onur Boyman (Department of Dermatology, University Hospital Zurich) and were cultured in Keratinocyte serum free medium (K-SFM; Invitrogen), supplements (EGF Human Recombinant and Bovine Pituitary Extract; Invitrogen), L-glutamine and antibiotics as described [21]. Normal human dermal fibroblasts (NHDF) were obtained from PromoCell GmbH (Heidelberg, Germany). Human microvascular endothelial cells (HMEC-1) were obtained as a gift from Dr. Therese Resink (Department of Biomedicine, University Hospital Basel) and were cultured in Dulbecco’s modified Eagle’s medium (DMEM; Invitrogen) supplemented with 5% FBS (Sigma-Aldrich) and antibiotics. Human lung fibroblasts (MRC-5) were obtained as a gift from Dr. Giancarlo Marra (IMCR, Zurich) and were originally obtained from Coriell Cell Repositories (Camden, NJ) and cultured in MEM medium (Invitrogen), containing 15% FBS, L-glutamine and antibiotics. ZT-821 cells (primary renal cells) were established in our lab from a single cell suspension of healthy kidney tissue. The NHDF and ZT-821 cells were cultured in RPMI 1640 medium (Invitrogen) containing 10% FBS (Sigma-Aldrich), L-glutamine and antibiotics.

### Treatment of cells

#### Ionizing radiation

Cells and fresh tumour biopsies were exposed to γ-radiation from a ^60^Co source. The doses of irradiation used are described in each experiment.

#### Hyperthermia

Cells were incubated at 42°C for 1 h or 5 h and cultured subsequently for 72 h at 37°C. The treated and non-treated cells were then processed for analysis by RT-qPCR.

#### Hypoxia

Cells were incubated under hypoxic conditions (1% O_2_ vol/vol) in a hypoxic workstation (InVivoO_2_-400, Ruskinn Technology, Leeds, UK) for different time points (8 h, 48 h or 72 h). The treated and non-treated cells were then processed for analysis by RT-qPCR.

#### Genotoxic stress

Cells were treated at 37°C with 1 µg/mL cisplatin (Sigma-Aldrich Corp. St. Louis, MO) for 24 h or with 15 µM etoposide (Sigma-Aldrich) for 1 h or with 150 µM bleomycin (Sigma-Aldrich) for 1 h. This time point was considered as T_0_. At the end of the treatment, the medium was replaced with fresh culture medium without the drug. The cells were then cultured for additional 72 h at 37°C. Control cultures were treated under similar experimental conditions in the absence of the drug.

#### Small-molecule protein kinase inhibitors

A stock concentration of 10 mM in 100 % dimethyl sulfoxide (DMSO) of specific inhibitors for ataxia telangiectasia mutated (ATM), KU 55933 (Tocris bioscience, Ellisville, MO) and DNA-dependent protein kinase catalytic subunit (DNA-PKcs), NU7441 (Tocris bioscience) were diluted to a working concentration of 10 µM in 1% DMSO. The inhibitors were added 1 h before irradiation, and were left in the culture throughout the experiment. Control cultures were treated under similar experimental conditions in the absence of the drug with 1% DMSO.

### Tumor biopsies

Biopsies were obtained from the University Hospital Zurich. All patients underwent surgery as part of their standard treatment and signed the informed consent. The ethics committee "Ethical committee of the canton of Zurich" specifically approved this study (Study No: EK-1017). Immediately upon resection, biopsies were cut into multiple pieces of 2-4 mm^3^. The pieces were randomized in two batches and put into culture medium. One batch was irradiated with a single dose of 20 Gy, whereas the other batch served as control. The tumor pieces were cultured for 72 h following *ex vivo* radiation and the gene expression was determined by RT-qPCR analysis. Paired biopsies from sarcoma before and after irradiation were collected for another purpose and therefore the time points of collection after radiotherapy varied between 2-6 weeks. The tumors were irradiated *in vivo* with a linear accelerator and received a dose of 50-64 Gy.

### RNA extraction and preparation of cDNA

Total RNA was extracted using the RNeasy Mini Kit (Qiagen, Valencia, CA) and was subsequently digested with DNAse I (Invitrogen). The concentration and purity was evaluated using the NanoDrop ND-1000 spectrophotometer (NanoDrop Technologies, Wilmington, DE). 150 ng of RNA was reverse transcribed using the high-capacity cDNA Reverse Transcription Kit (Applied Biosciences, Foster City, CA). The cDNA was either used immediately for RT-qPCR reactions or stored at -20°C until use. All kits were used according to the manufacturer’s instructions.

### RT-qPCR

The expression of CT-antigens and MHC-I was analyzed using TaqMan gene analysis primers and TaqMan 1x universal master mix (Applied Biosystems) on a RotoGene cycler (Corbett Research, Sydney, NSW). The reaction mixture (10 µL) consisted of 1 µL cDNA, 3.5 µL water, 0.5 µL primer and 5 µL TaqMan 1x universal master mix. The following cycle conditions were used: 2 min 50°C, 10 min 95°C, 45 cycles of 15 s at 95°C, 1 min 60°C. The change in expression levels of CT-antigens and MHC-I is given as the fold increase in expression by comparing the delta-Ct values of the treated to that of the non-treated samples after normalization to 18S rRNA. Ct values > 38 cycles were interpreted such that the gene is not expressed and a fold expression < 2 was considered as no change. Each of the breast cancer and osteosarcoma cell lines were tested in two independent experiments and the lung carcinoma and the prostate carcinoma cell lines in one. Each sample was loaded in duplicates/triplicates for each of the RT-qPCR experiments.

### Flow cytometry

Cells were stained with PE-labeled anti-HLA-A,B,C (clone G46-2.6, 1∶100), FITC-labeled anti-β2-microglobulin (clone TU99, 1∶100) (BD Pharmingen, San Diego, CA) and propidium iodide (PI; Sigma). Samples were measured with a FACS Calibur (BD) and analyzed with FlowJo software (Treestar) after gating on live (PI-negative) cells. Appropriate isotype controls were used.

### Immunohistochemistry

Formalin-fixed, paraffin-embedded paired tissue sections obtained from sarcoma patients before and after irradiation were stained with mouse anti-human monoclonal antibodies against CD4 (clone 1F6, 1∶30, ZYMED Laboratories Inc.), CD8 (clone C8/114B, 1∶100, DAKO A/S, Carpinteria, CA), granzyme B (clone Gr B-7, 1∶25, DAKO A/S), MHC-I (clone C21, 1∶1000, RDI Research Diagnostics, Inc.), CT7 (clone CT7-33, 1∶80, DAKO A/S), CT10 (rabbit polyclonal, 1∶500, ProteinTech Group, Inc.), NY-ESO-1 (clone E978, 1∶50, ZYMED) and Perforin (clone 5B10, 1∶20, Novocastra Laboratories Ltd). Sections were counterstained with hematoxylin, dehydrated and mounted. All sections were stained either with the Ventana Benchmark automated staining system (Ventana Medical Systems, Tucson, AZ) using Ventana reagents for the entire procedure for NY-ESO-1, CT7, granzyme B, CD4, CD8 and perforin and BondMax (Vison BioSystems, Newcastle upon Tyne, UK) for CT10 and MHC-I. UView (Ventana) or Refine DAB (Vision BioSystems) were used as chromogens against the primary antibodies. Images of the stained sections were acquired on Zeiss-Axiovert 200 M (Carl Zeiss Light Microscopy Göttingen, Gearmany) inverse microscope using Carl Zeiss Axiovision CD28 imaging system. The stainings were scored on a scale of 0 to 5 for the expression of NY-ESO-1, MAGE-C1/CT7, and MAGE-C2/CT10 as a percentage, based on the number of positive cells expressing the antigen to the total number of cells in a high power field (HPF) using a 40X objective lens. The scoring for MHC-I expression was done based on the intensity of staining. The tumor infiltrates were calculated as the number of cells expressing CD4, CD8 and granzyme per HPF using a 40X objective. Each stained section was evaluated in five different regions (for detailed list of scores see **Table S4**). Three individuals performed the scoring blindly and independently for the MHC-I stainings and two individuals performed the scoring blindly for the other stainings.

### Immunofluorescence

Cells (10’000-20’000 cells in 1 mL medium) were plated on compartmented culture slides (BD Biosciences) to adhere overnight. Cells were then fixed with 4% paraformaldehyde for 15 min at room temperature, permeabilized with 0.1% TritonX-100 (Sigma-Aldrich) for 5 min at room temperature, followed by blocking with 10% BSA/PBS for 30 min at room temperature. The cells were then incubated with monoclonal mouse antibodies against NY-ESO-1 (clone E978, 1∶500, Invitrogen) or MAGE-C1/CT7 (clone CT7-33, 1: 500, Dako) in 10% BSA/PBS for 1 h at room temperature in the dark, followed by incubation with FITC- labeled secondary antibody goat anti-mouse IgG1 (Poly4053, 1∶2000, Biolegend) for 15 min at room temperature in the dark. The slides were counterstained with 4',6'-diamidino-2-phenylindole hydrochloride (DAPI, 1∶500) for 2 minutes in the dark and inspected using a Zeiss-Axiovert 200 M inverse microscope and a Carl Zeiss Axiovision CD28 imaging system.

### Western blot analysis

Irradiated and non-irradiated cells were checked for the protein expression using monoclonal mouse antibodies against NY-ESO-1 (clone E978, 1∶500, Invitrogen) or MAGE-C1/CT7 (clone CT7-33, 1: 500, Dako) and β-actin (clone 4i374, 1∶8000, ***Santa Cruz*** Biotech, CA). The effect of genotoxic agents and DNA-PKcs and ATM inhibition, on the γ-irradiation induced gene expression in cells was evaluated by using antibodies against pS2056-DNA-PKcs (rabbit polyclonal to phospho S2056-DNA-PKcs, 1∶300, Abcam Inc, MA), FANC-D2 (clone Fl17, 1∶200, ***Santa Cruz*** Biotech, CA), pS1981-ATM (clone EP1890Y, 1∶5000, Eitomics, CA) and pS15-p53 (clone 16G8, 1∶1000, Cell Signaling Technology, MA). Followed by incubation with the secondary antibody, polyclonal goat anti-mouse IgG1 HRP (Poly4053, 1∶10000, Biolegend) or polyclonal goat anti-rabbit HRP (1∶10000, Abcam). ECL reagent (Amersham, UK) was used as luminescence substrate.

### CD107a Degranulation Assay

Recognition of cells expressing NY-ESO-1 following irradiation by antigen-specific CD8^+^ T cells was tested using the CD107a degranulation assay. Three x 10^5^ NY-ESO-1_157-165_/HLA-A2-specific cloned CD8^+^ T cells (generated as described [22]) were incubated with 10^6^ CFSE-labeled (1 µM) HLA-A2^+^ MDA-MB-469 cells – that were or were not irradiated with 20 Gy 72 h earlier – in a 96-well microtiter roundbottom plate in a final volume of 200 µL RPMI + 10% FCS and antibiotics in the presence of PE-labeled anti-CD107a (1∶20, BioLegend, SD, California). As positive control, MDA-MB-469 cells were loaded with 10^-6^ M NY-ESO-1_157-165_ peptide. Two independent T cell clones (2A7 and 2B5) were used and all cultures were performed in duplicate. The incubation was performed at 37°C for 4 h. The cells were collected and washed in 2 mL FACS-buffer (FB) followed by staining with pacific blue-labeled anti-CD8 (BioLegend) in 50 µL FB for 30 min at 4°C. The cells were washed once with 2 mL FB and resuspended in 200 µL FB. Samples were measured on CyAn ADP9 (Beckman Coulter Inc, Fl, USA) and analyzed with FlowJo software (Treestar).

## Results

### *In vitro* γ-radiation up-regulates transcripts of CT-antigens and MHC-I molecules

**Figure 1:**
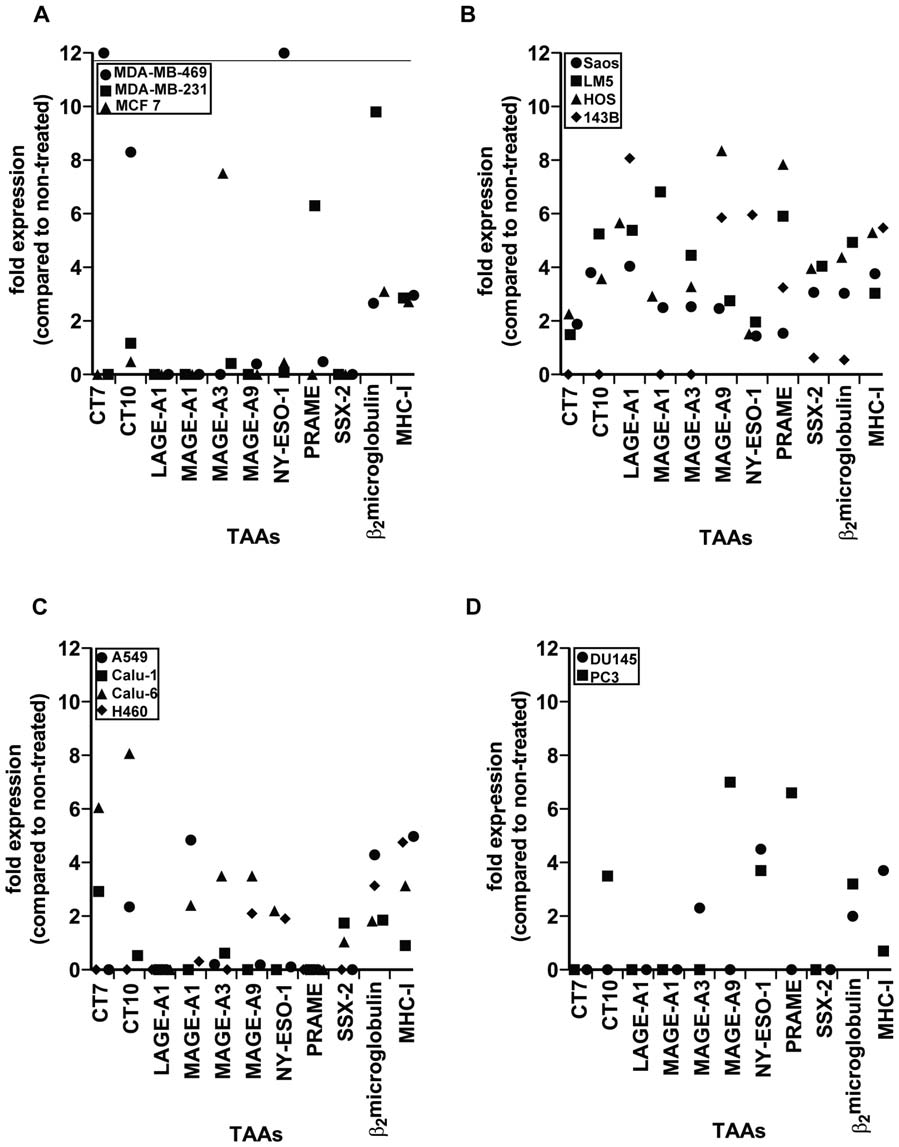
γ-radiation up-regulates CT-antigens and MHC-I molecules on the mRNA level. Established cancer cell lines were exposed to single dose irradiation of 20 Gy and the mRNA expression of CT-antigens and MHC-I was determined 72 h later by RT-qPCR. (A) breast cancer cell lines, (B) osteosarcoma cell lines, (C) lung cancer cell lines, (D) prostate cancer cell lines. All Ct values are normalized to 18S rRNA and the data are presented as the fold increase of expression in irradiated compared to the corresponding untreated samples. Because fold expression can’t be calculated for genes that are *de novo* expressed upon irradiation, we put the symbols in such cases above the thin horizontal line in (A).

Breast, osteosarcoma, lung and prostate cancer and normal primary cells were exposed to a single-dose radiation of 20 Gy, after 72 h the cells were harvested and analyzed for the expression of CT-antigens and MHC-I by RT-qPCR. Most of these cancer cell lines expressed undetectable or very low levels of CT-antigens under standard culture conditions. In contrast, γ-radiated cells showed *de novo* or up-regulated expression of various CT-antigens in a randomized fashion (**Figure 1A-D**). In a panel of the four cancer cell line types, γ-radiation was found to have the most profound effect on the breast cancer and osteosarcoma cell lines and the least on the prostate cancer cell lines. The up-regulation of MHC-I and CT-antigens upon γ-radiation seems to be a specific feature of malignant cells, as this was not observed in any of the normal primary cells that we tested here (ZT-812, human foreskin keratinocytes, HMEC-1, MRC-5, NHDF and MCF 10A, **Figure S1A, S1B**). The melanoma cell line SK-MEL-37 is known to express all tested CT-antigens under normal culture conditions and was therefore included as the positive control. Irradiation of this cell line resulted in a clear up-regulation of all the CT-antigens and MHC-I (data not shown). We detected an increased expression of CT-antigens and MHC-I as early as 24 h after irradiation in some cases and a steady increase in gene expression was observed up to 96 h after irradiation. As we observed substantial cell death at 96 h after irradiation, we performed all further analyses at 72 h after irradiation. As radiotherapy can be given as a single high dose (20 Gy) or as fractionated doses, we irradiated selected cell lines (the breast cancer cell lines MDA-MB-469 and MCF7, as well as the osteosarcoma cell lines Saos, HOS, LM5 and 143B, with 2 Gy per day on 10 consecutive days. This protocol resulted in similar change of CT-antigen and MHC-I expression as observed with a single dose of 20 Gy (**Figure S2A, S2B**). A single dose of 20 Gy was used in all further experiments.

### *In vitro* γ-radiation up-regulates CT-antigens and MHC-I molecules at the protein level

**Figure 2:**
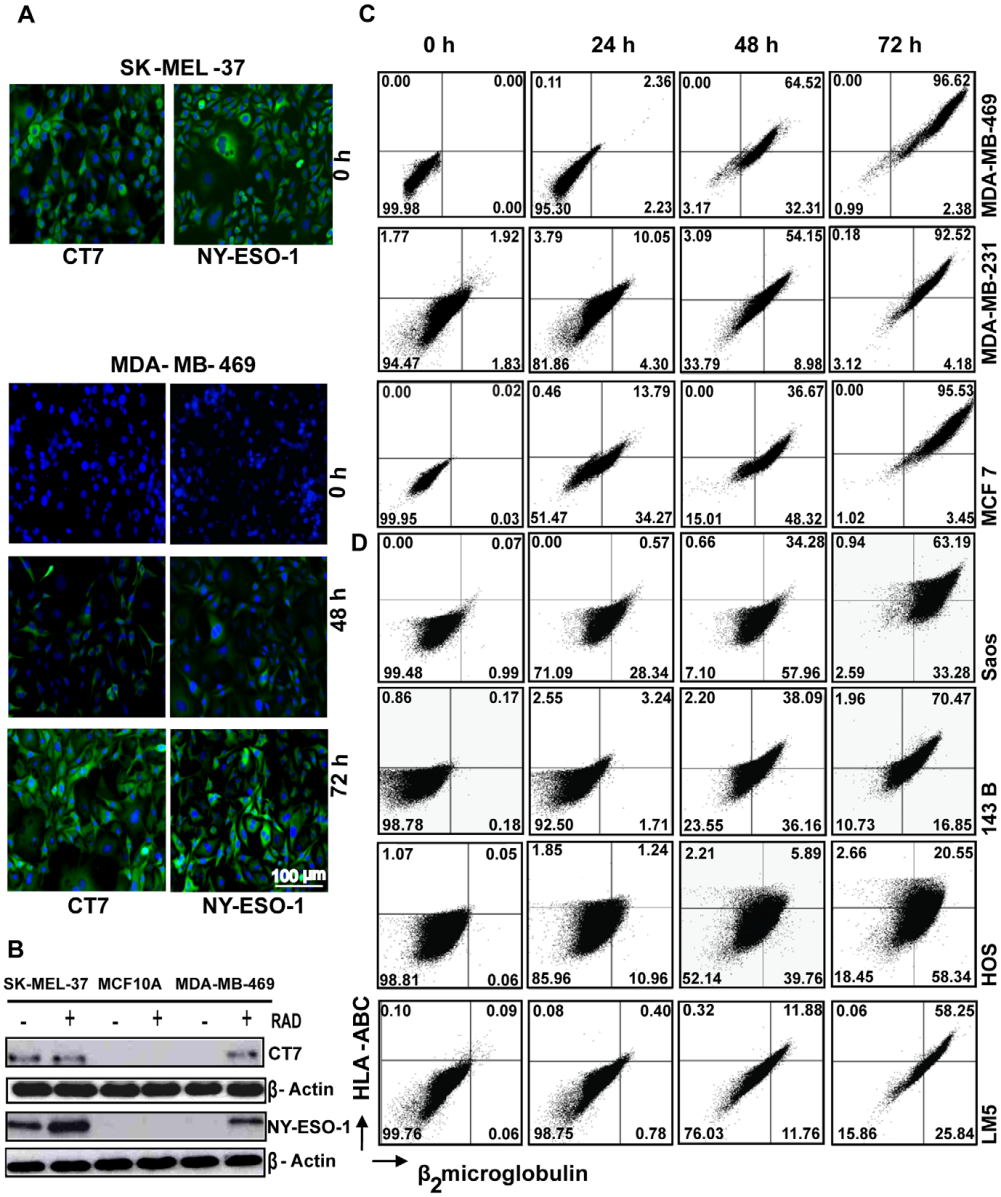
γ-radiation up-regulates CT-antigens and MHC-I molecules on the protein level. (A) Immunofluorescence of MDA-MB-469 and SK-MEL-37 cells exposed to a single dose irradiation of 20 Gy and stained with antibodies against NY-ESO-1 and CT7 at different time points after irradiation. (B) MDA-MB-469, SK-MEL-37 and MCF 10A cell lines were exposed to a single dose of 20 Gy and the expression of NY-ESO-1 and CT7 was analyzed by Western blotting 72 h later. (C) breast cancer and (D) osteosarcoma cell lines were exposed to single dose irradiation of 20 Gy and the expression of HLA-ABC and β_2_microglobulin was quantified at different time points after irradiation by flow cytometry. RAD: indicates γ-radiation.

To confirm the irradiation-induced expression of CT-antigens on the protein level, we stained irradiated and non-irradiated MDA-MB-469 cells with antibodies against NY-ESO-1 and CT7 and analyzed the expression of CT7 and NY-ESO-1 at different time points after irradiation by immunofluorescence microscopy and by Western blotting. The melanoma cell line SK-MEL-37 constitutively expresses NY-ESO-1 and CT7 and was used as positive control. We observed the *de novo* expression of both the CT-antigens upon irradiation that increased with time (**Figure 2A, 2B**), thus confirming the data obtained by RT-qPCR. Irradiation of the normal breast cell line MCF10A did not result in increased NY-ESO-1 or CT7 protein levels (**Figure 2B**). To study the effect of γ-radiation on the surface expression of MHC-I at the protein level, we irradiated multiple cancer cell lines (breast carcinoma and osteosarcoma) and normal primary cells of different origins, followed by flowcytometric detection of surface MHC-I, and observed that irradiation resulted in a time-dependent increase of MHC-I surface expression on different cancer cell lines (**Figure 2C, 2D**) but not in the normal primary cells **(Figure S1B).**

### Irradiation of cancer cells enhances T cell recognition *in vitro*

**Figure 3:**
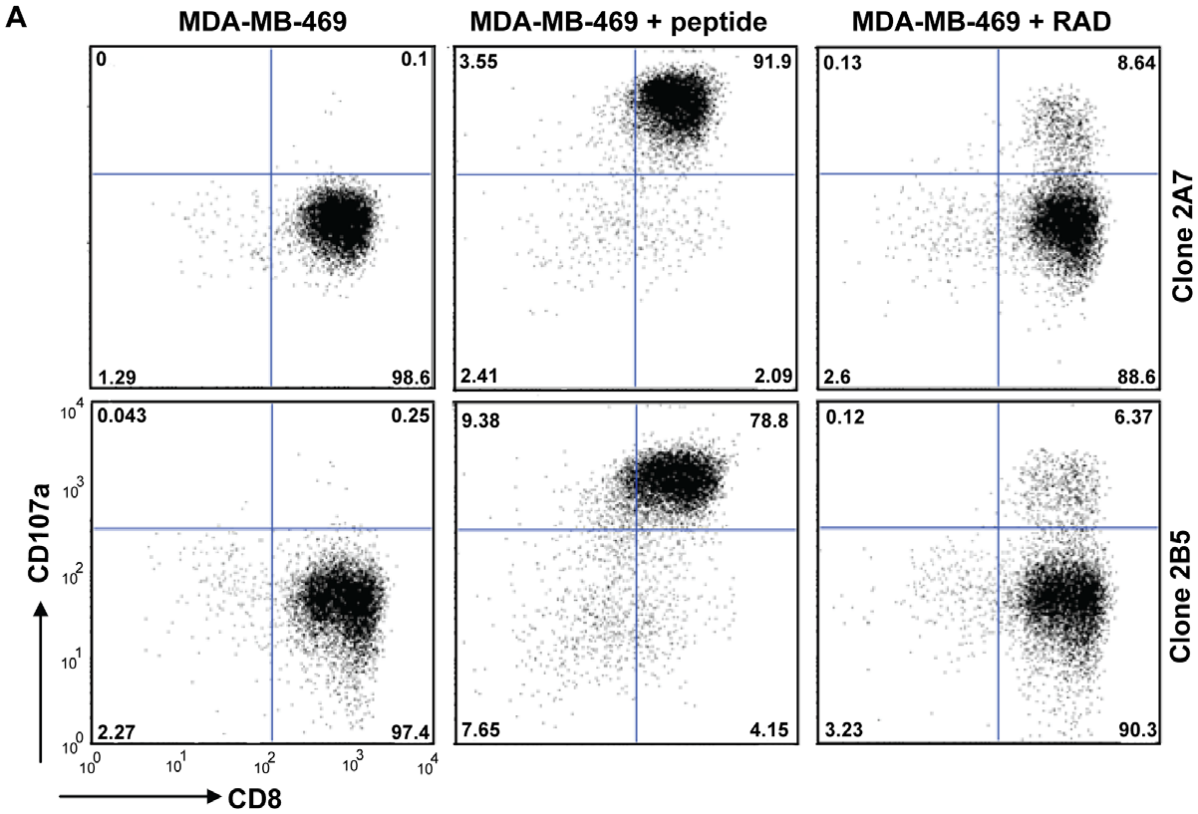
Irradiation induced enhances T cell recognition of cancer cells. 10^6^ CFSE-labeled HLA-A2+ MDA-MB-469 breast cancer cells were irradiated or not with a single dose of 20 Gy and were incubated 72 h later with 3×10^5^ NY-ESO-1_157-165_/HLA-A2-specific CD8+ T cell clones (clone 2A7 and clone 2B5) in the presence PE-labeled anti-CD107a-PE for 4 h at 37°C. The cells were then stained with pacific blue-labeled anti-CD8- for 30 min at 4°C. Peptide-loaded MDA-MB-469 cells were used as positive control. All cultures were performed in duplicate. The degranulation was measured as percentage CD107a^+^ cells of CD8^+^ cells after gating on CFSE-negative cells by flow cytometry.

In order to determine whether irradiation-induced up-regulation of CT-antigens and MHC-I results in increased recognition by antigen-specific CD8^+^ T cells, we measured degranulation [23]–[24] of NY-ESO-1_157-165_/HLA-A2-specific cloned CD8^+^ T cells upon incubation with irradiated and non-irradiated HLA-A2^+^ MDA-MB-469 breast cancer cells. MDA-MB-469 cells are negative for NY-ESO-1, but become positive upon irradiation (**Figure 1A** **,** **2A, 2B**). We found that only irradiated MDA-MB-469 cells induced degranulation of two independent NY-ESO-1_157-165_-specific CD8^+^ T cell clones (2A7, 2B5) (**Figure 3**). Irradiation did not further increase the degranulation when peptide-loaded MDA-MB-469 cells were used (data not shown), indicating that not the amount of MHC-I but the amount of NY-ESO-1_157-165_ presented by MHC-I was the limiting factor in unirradiated MDA-MB-469 cells, which fits the fact that irradiation induced *de novo* expression of NY-ESO-1 in MDA-MB-469 cells (**Figure 1A** **,** **2**).

### *Ex vivo* irradiation of fresh tumor biopsies induces up-regulation of CT-antigens and MHC-I

**Figure 4:**
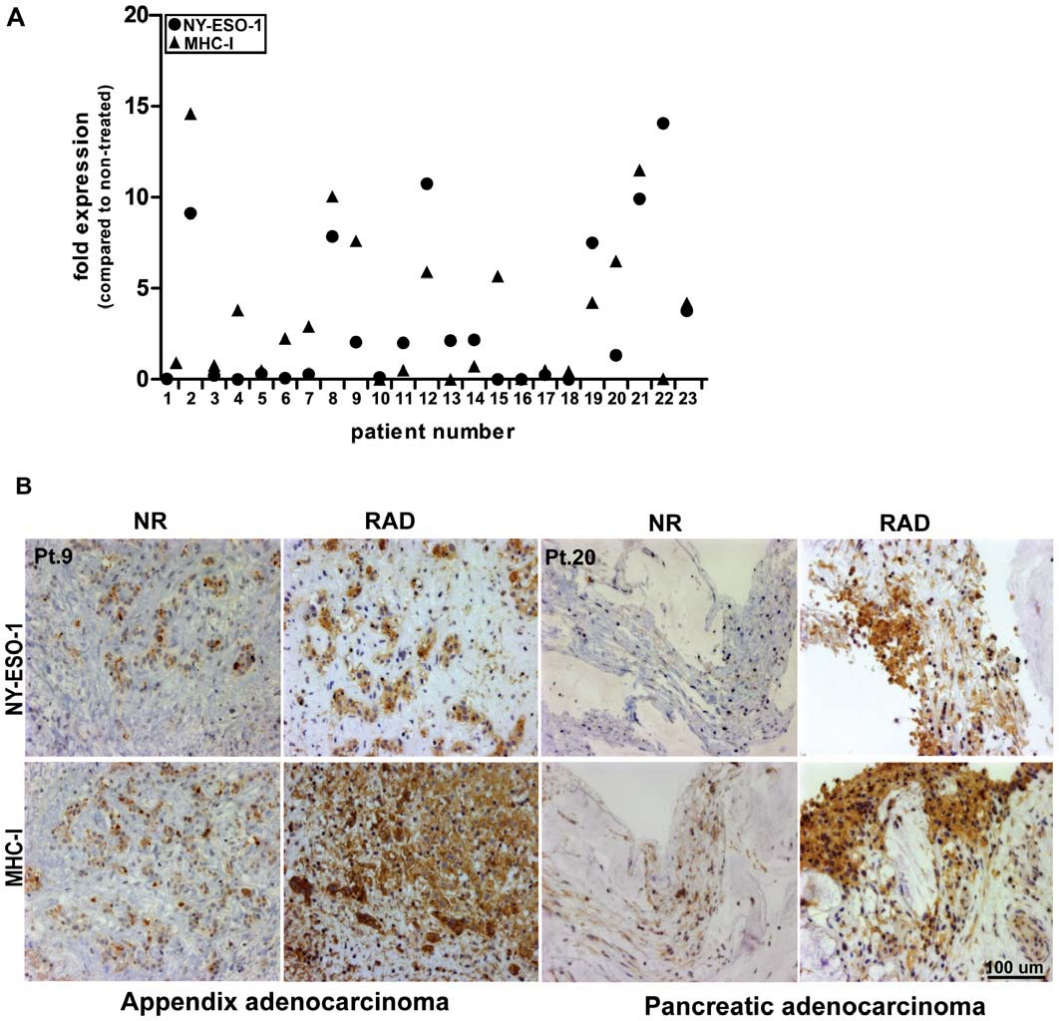
*Ex vivo* irradiation up-regulates CT-antigens and MHC-I in fresh tumor biopsies. Fresh tumor explants from different cancer patients (n = 23) were irradiated or not with single-dose of 20 Gy. The diagnosis of the individual patients is shown in supplementary Table S1. (A) After 72 h, the irradiated and control explants were analyzed for the expression of NY-ESO-1 and MHC-I by RT-qPCR. (B) Representative sections (patient number 9 and 20) depicting the expression of NY-ESO-1 and MHC-I by immunohistochemistry (20X magnification). NR indicates non-radiated and RAD indicates corresponding irradiated sections.

To expand our findings to clinically relevant material, we collected fresh tumor biopsies from 23 different cancer patients, cut those into at least 50 small pieces, and randomized them into two experimental groups. We irradiated one group of biopsies with 20 Gy, whereas the others served as control, and analyzed the expression of CT-antigens and MHC-I 72 h later by RT-qPCR and immunohistochemistry. These results confirmed the findings we obtained with cancer cell lines, that γ-radiation often induced increased expression of CT-antigens and/or MHC-I (**Figure 4A, 4B** **, Table S1**). Importantly, our results suggest that heterogeneous expression of CT-antigens and/or MHC-I can become more homogeneous upon radiotherapy, which obviously supports immunological control of the tumor.

### *In vivo* irradiation of human sarcoma results in increased expression of CT-antigens and MHC-I and in lymphocyte infiltration

**Figure 5:**
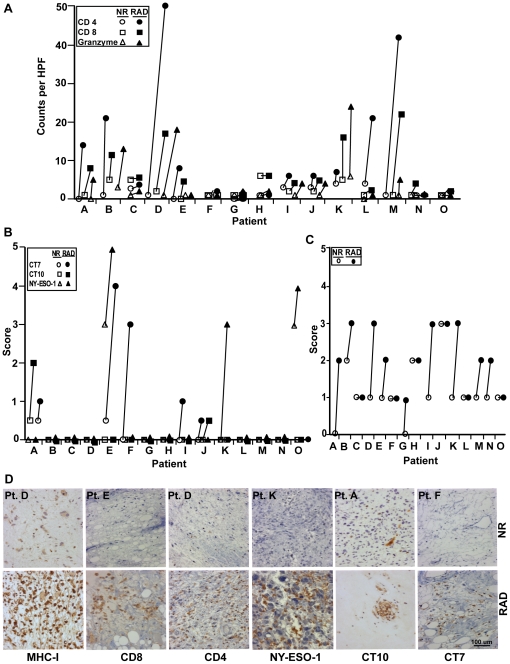
Radiotherapy induced expression of CT-antigens and MHC-I and lymphocyte infiltration in sarcoma patients. Paraffin-embedded paired tissue sections obtained from sarcoma patients (n = 15) before and after irradiation were analyzed by immunohistochemistry for (A) presence of CD8^+^, CD4^+^ and granzyme^+^ cells, (B) expression of CT7, NY-ESO-1 and CT10 and (C) MHC-I expression. The CT-antigens were scored as the mean percentage of live cells expressing the antigen to the total number of cells in five high power fields (40X objective). The infiltration by lymphocytes was taken as the mean by counting the number of cells expressing CD4, CD8 and granzyme in five high power fields, and MHC-I was scored based on the intensity of the staining. (D) Representative sections showing the expression of CT-antigens and MHC-I and infiltration of lymphocytes before and after radiotherapy by immunohistochemistry. Depicted are: patient F for the expression of CT7, patient A for CT10, patient K for NY-ESO-1, patient D for CD4 and MHC-I and patient E for CD8 expression. NR indicates non-radiated and RAD indicates corresponding irradiated sections. Pt: indicates patient number. Patient information is listed in supplementary Table S2.

Finally, we compared the expression of CT-antigens, MHC-I and the infiltration by lymphocytes in 15 paired paraffin-sections obtained from sarcoma patients before and after radiotherapy by immunohistochemistry. We found that radiotherapy resulted in substantial up-regulation or *de novo* expression of CT-antigens and/or MHC-I molecules, which was accompanied by an increased infiltration by lymphocytes and granzyme expression in 7/15 and 12/15 samples, respectively (**Figure 5A-D** **, Table S2, S3**).

### Other forms of stress have no impact on the expression of CT-antigens or MHC-I *in vitro*

Stress and environmental alterations induce changes in the transcriptional profile in order to cope with those assaults [25], [26], [27]. As irradiation induces DNA-damage, we investigated whether the up-regulated expression of MHC-I and CT-antigens would also occur after exposure of cell lines to other treatments that induce DNA damage such as cisplatin, etoposide and the radiomimetic drug bleomycin. MDA-MB-469 and SK-MEL-37 cells were treated separately with each of the DNA-damaging agents and RNA levels were monitored at different time points following treatment. Our results show that none of these agents up-regulated the expression of CT-antigens and MHC-I (**Figure S3B-D**). However, the hallmark genes pS15-p53 and FANC-D2 were up-regulated following treatment, indicating that these agents induced stress at the concentration used (**Figure S3A**). Furthermore, we tested whether other types of cancer-related stress, including elevated temperatures or low oxygen levels, induced increased expression of CT-antigens and/or MHC-I. We found that none of these treatments impacted on the expression of CT-antigens and MHC-I whereas the signature gene CA9 was up-regulated (**Figure S4A, S4B**). Together, these results suggest that the increased expression of CT-antigens and MHC-I by cancer cell lines is a specific response to γ-radiation and does not occur after exposition to other inducers of various stress response pathways.

### The ATM and DNA-PK signaling pathways are dispensable for γ-radiation-induced expression of CT-antigens and MHC-I

**Figure 6:**
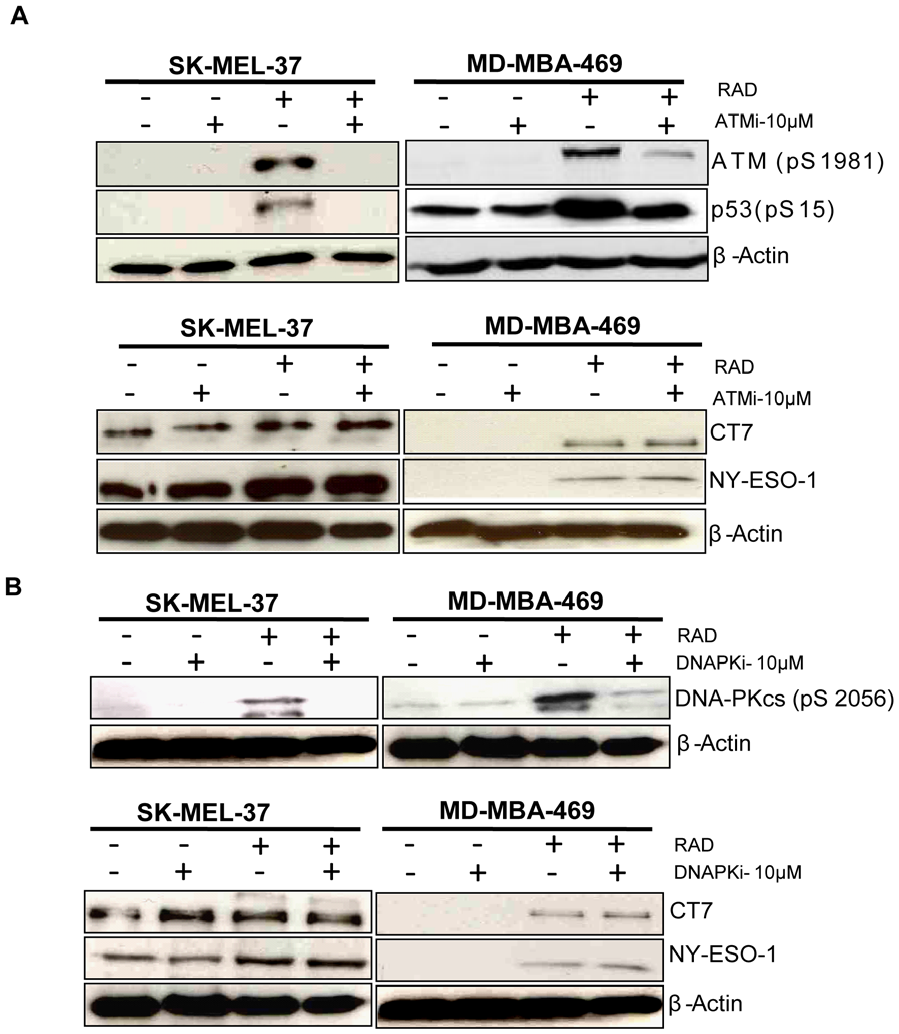
The ATM and DNA-PK signaling pathways are dispensable for γ-radiation induced expression of CT-antigens and MHC-I. SK-MEL-37 and MDA-MB-469 cells were irradiated or not in the presence or absence of inhibitors specific for ATM (ATMi) and DNA-PKcs (DNAPKi) and whole cell extract was analyzed by Western blotting. (A) Detection of phosphorylated ATM, p53, CT7 and NY-ESO-1 and (B) phosphorylated DNA-PK, CT7 and NY-ESO-1. Anti-β-actin specific antibodies served as loading controls.

The activation of DNA-damage repair checkpoint pathways as a response to genotoxic insult helps to maintain the genomic integrity in mammalian cells [28]. DNA damage triggers the activation of various serine/threonine protein kinases, which constitute the primary transducers in the signaling cascade and of which, ataxia telangiectasia mutated (ATM) and DNA-dependent protein kinases (DNA-PKcs) are of utmost importance [29]. The ATM protein kinase is a critical intermediate in a number of cellular responses to **γ-**radiation and other forms of stress [30]. We thus investigated whether the up-regulation of CT-antigen and MHC-I expression in response to γ-radiation depends on the activation of ATM and/or DNA-PKcs. We irradiated MDA-MB-469 and SK-MEL-37 cells with a single dose of γ-radiation (20 Gy) in the presence or absence of specific inhibitors of ATM (KU55933) and DNA-PK (NU7441). 10 µM of the inhibitor was added 1 h before irradiation, and was left in the culture throughout the experiment. We observed that ATM and DNA-PK as well as p53 were phosphorylated in response to γ-radiation, which was prevented by the specific inhibitors (**Figure 6A, 6B**). However, neither ATM- nor DNA-PK inhibition prevented the up-regulation of CT-antigen and MHC-I expression (**Figure 6A, 6B** **, S5A, S5B)**. These results indicate that the ATM and DNA-PK DNA-damage signaling pathways are not crucially involved in the up-regulation of CT-antigen and MHC-I expression upon γ-radiation**.**

## Discussion

Radiotherapy is one of the most widely used and successful cancer treatments to date [31]. Recent studies described that γ-radiation leads to a plethora of alterations in the tumor cells [32], including the *de novo* synthesis of particular proteins and the up-regulation of MHC-I expression [1], [32], [33]. In addition, irradiated tumors are often more infiltrated by leukocytes than the non-irradiated tumors and studies using preclinical models showed that the therapeutic success of high-dose irradiation depends on adaptive immunity [34]. We therefore hypothesized that the expression of a specific class of TAAs, the so-called CT-antigens, is induced by irradiation, thus making tumors more susceptible to recognition by effector T cells. In addition the *de novo* expression of highly immunogenic CT-antigens may result in tumor-specific immune responses that are not yet compromised by mechanisms of central [35] or peripheral tolerance. Because cancer cells can only be recognized by CD8^+^ T cells when they express MHC-I and because tumors often express very low levels of MHC-I or are even negative, radiation-induced up-regulation of MHC-I may further support immune recognition. We observed that irradiation induced *de novo* or up-regulated expression of various CT-antigens and MHC-I in a randomized fashion and independent of the tissue origin of the malignancy. We confirmed this effect using established cancer cell lines, fresh *ex vivo* irradiated tumors and paired biopsies from sarcoma patients before and after radiotherapy. Studies have shown that the radiation-induced up-regulation of MHC-I makes the tumor cells more susceptible to lysis by CTL *in vitro* [1], [36]. We confirm these data here and show that irradiation of tumor cells not only up-regulates the expression of CT-antigens and MHC-I but also increases their recognition by CD8^+^ T cells. The up-regulated expression of CT-antigens and MHC-I seems specific for γ-radiation, as similar changes in gene expression were not observed upon other treatments that induce DNA-damage, upon hypoxia or hyperthermia. We could not identify the molecular mechanism underlying radiation-induced up-regulation of CT-antigens and MHC-I expression, but we excluded the involvement of the ATM or DNA-PK signaling pathways. Previous studies have shown that the expression of CT-antigens is regulated through demethylation of their promoter CpGs [37]. In addition, all CT-antigen genes that are expressed in tumors or testis can be induced *in vitro* by DNA demethylation [38]. However, when we compared the methylation status of CpGs in the promoter region of NY-ESO-1 of irradiated and non-irradiated MDA-MB-469 and MCF 7 cells, we found no difference (data not shown). This result suggests that the up-regulated expression of CT- antigens and MHC-I upon γ-radiation is regulated through another pathway than demethylation of CpG islands in the promoter region. However, as we performed this experiment only with a limited number of cell lines and we analyzed only one CpG region in the promoter of NY-ESO-1, we can’t fully exclude that other CpG regions may have been hypomethylated following irradiation.

Our data strongly suggests that irradiation supports immunological control of tumors through the expression of novel tumor-associated antigens to which the immune response presumably is not compromised. In addition, the concomitant up-regulation of MHC-I expression makes irradiated tumor cells more susceptible to tumor-specific CTL. It is currently not known whether the increased infiltration by CD8^+^ T cells upon irradiation is a direct effect of increased local expression of cognate peptide/MHC-I complexes or whether other factors such as local inflammation or changes in vasculature contribute.

It may be interesting to combine radiotherapy with immunization to maximally exploit the changes induced by irradiation. However, the fact that it seems unpredictable as to which CT-antigens will show *de novo* or up-regulated expression makes it difficult to choose the correct antigen for immunization, at least in those cases where tumor biopsies are not available. Nevertheless, the combination of radiotherapy with treatments that generally stimulate the immune system such as the blockade of co-inhibitory interactions (CTLA-4, PD-1) or mediators (IL-10, TGF-β, IDO) or activation of dendritic cells by innate stimuli may further improve the efficacy of radiotherapy.

## Supporting Information

Figure S1 **γ-radiation does not effect the expression of CT-antigens and MHC-I molecules** ***in vitro*** **.** Normal primary cell cultures – HMEC-1, human foreskin keratinocytes, MCF-10A, MRC-5, NHDF and ZT-812, were exposed to a single dose irradiation of 20 Gy and the CT-antigen and MHC-I expression was determined at the (A) mRNA level by RT-qPCR, and (B) protein level by flow cytometry.
(TIF)

Figure S2 **Fractionated γ-radiation results in a time-dependent up-regulation of CT-antigens and MHC-I molecules** ***in vitro*** **.** Established cancer cell lines were exposed to fractionated irradiation of 2 Gy on 10 consecutive days to obtain a cumulative dose of 20 Gy. (A) breast cancer cell lines, (B) osteosarcoma cell lines. All Ct values are normalized to 18S rRNA and the data are presented as the fold increase of expression in irradiated (at 24 h, 48 h and 72 h from the last dose of irradiation) compared to the corresponding untreated samples.
(TIF)

Figure S3 **Genotoxic stress has no impact on the expression of CT-antigens or MHC-I** ***in vitro***. The breast cancer cell line MDA-MB-469 and the melanoma cell line SK-MEL-37 and the normal breast cell line MCF 10A were exposed to other forms of stress and gene expression was analyzed after 72 h treatment with DNA-damaging agents. (A) Treatment with DNA-damaging agents followed by immunoblotting to detect the activation of hallmark genes p53 and FANC-D2. The same samples were also subjected to RT-qPCR analysis for the expression of CT-antigens and MHC-I at different time points following treatment with (B) bleomycin, (C) cisplatin and (D) etoposide. All Ct values are normalized to 18S rRNA and the data are presented as the fold increase of expression in treated compared to the corresponding untreated samples.
(TIF)

Figure S4 **Other forms of stress have no impact on the expression of CT-antigens or MHC-I** ***in vitro***. MDA-MB-469 and SK-MEL-37 cells were exposed to (A) hyperthermia and (B) hypoxia and the gene expression following treatment was monitored at different time points by RT-qPCR analysis. All Ct values are normalized to 18S rRNA and the data are presented as the fold increase of expression in treated compared to the corresponding untreated samples.
(TIF)

Figure S5 **γ-radiation induced expression of CT-antigens and MHC-I is not dependent on the activation of ATM, DNA-PK signaling pathways.** The breast cancer cell line MDA-MB-469, the melanoma cell line SK-MEL-37 and the normal cell line MCF 10A were exposed or not to a single dose γ-radiation of 20 Gy in the presence or absence of specific inhibitors of the DNA-damage repair pathways (A) ATM or (B) DNA-PKcs, followed by RT-qPCR analysis for gene expression 72 h following irradiation. All Ct values are normalized to 18S rRNA and the data are presented as the fold increase of expression in treated compared to the corresponding untreated samples.
(TIF)

Table S1 **CT-antigens and MHC-I expression in fresh tumor biopsies following** ***ex vivo*** **radiation.** Fold increase in expression of CT-antigens and MHC-I in fresh tumor biopsies following 20 Gy *ex vivo* γ-radiation. All characters in bold represent up-regulation following *ex vivo* γ-radiation. ND: indicates non-detected. All Ct values are normalized to 18S rRNA and the data are presented as the fold increase of expression in treated compared to the corresponding untreated samples.
(DOC)

Table S2 **Increased expression of CT-antigens and lymphocyte infiltration in sarcoma patients after radiotherapy.** (A) Information of sarcoma patients treated with radiotherapy with immunohistochemical scores for T cell infiltration and expression of CT-antigens. All characters in bold represent up-regulation following radiotherapy. MPNST: malignant peripheral nerve sheath tumour, NOS: not otherwise specified. NR indicates non-radiated and RAD indicates corresponding irradiated sections.
(DOC)

Table S3 **Radiotherapy up-regulates the expression of MHC-I in sarcoma patients.** MHC-I expression in sarcoma patients following radiotherapy. All characters in bold represent up-regulation following radiotherapy. NR indicates non-radiated and RAD indicates corresponding irradiated sections.
(DOC)

Table S4 **Quantification of CT-antigen and MHC-I expression in sarcoma patients.**
(DOC)
