# Supplementary figures and images for: γ-Radiation Promotes Immunological Recognition of Cancer Cells through Increased Expression of Cancer-Testis Antigens In Vitro and In Vivo
Source: PLoS One. 2011 Nov 29;6(11):e28217. doi: 10.1371/journal.pone.0028217 (PMC3226680; doi:10.1371/journal.pone.0028217)

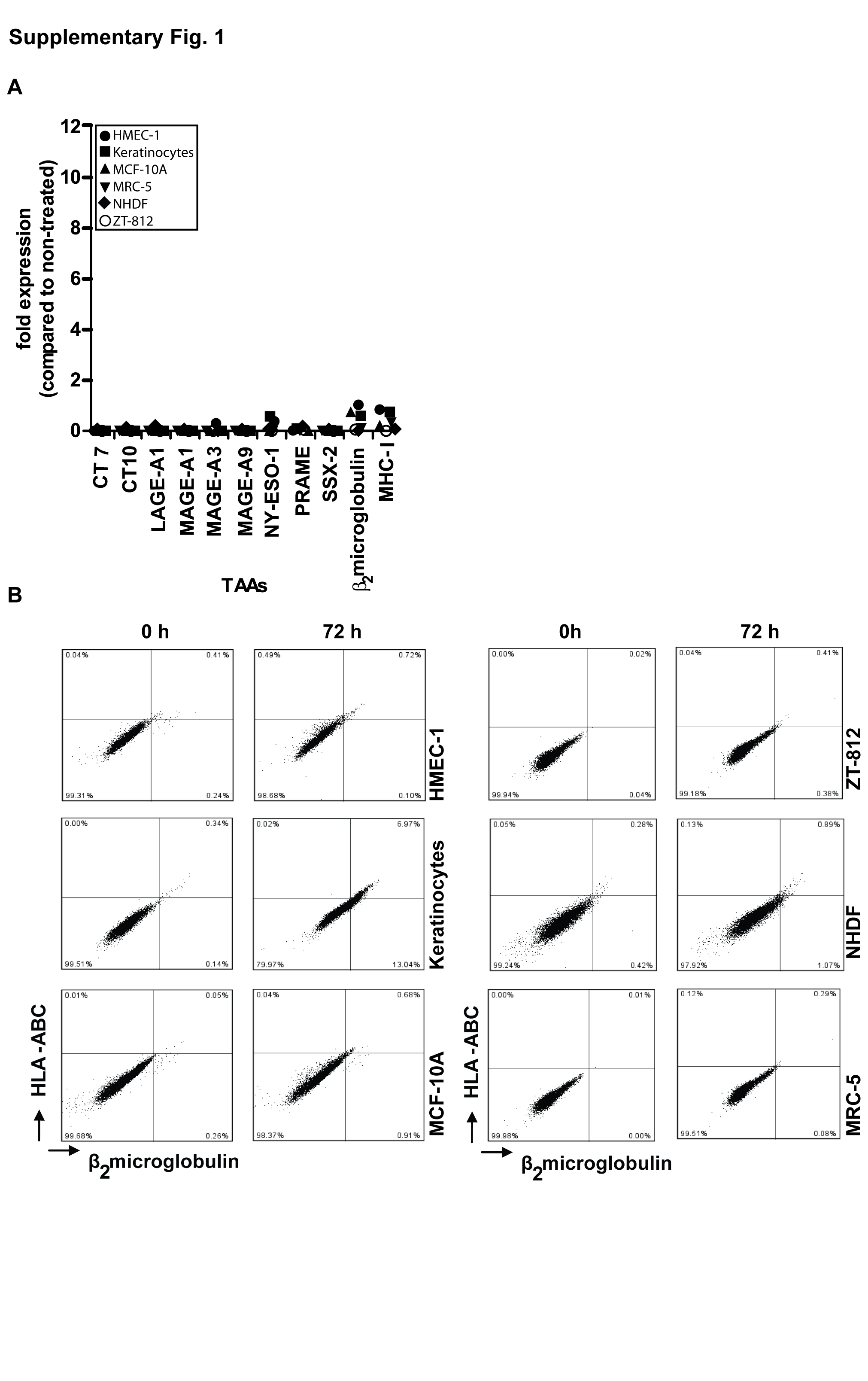

Supplement: Figure S1 — γ-radiation does not effect the expression of CT-antigens and MHC-I molecules in vitro . Normal primary cell cultures – HMEC-1, human foreskin keratinocytes, MCF-10A, MRC-5, NHDF and ZT-812, were exposed to a single dose irradiation of 20 Gy and the CT-antigen and MHC-I expression was determined at the (A) mRNA level by RT-qPCR, and (B) protein level by flow cytometry. (TIF) [file pone.0028217.s001.tif]

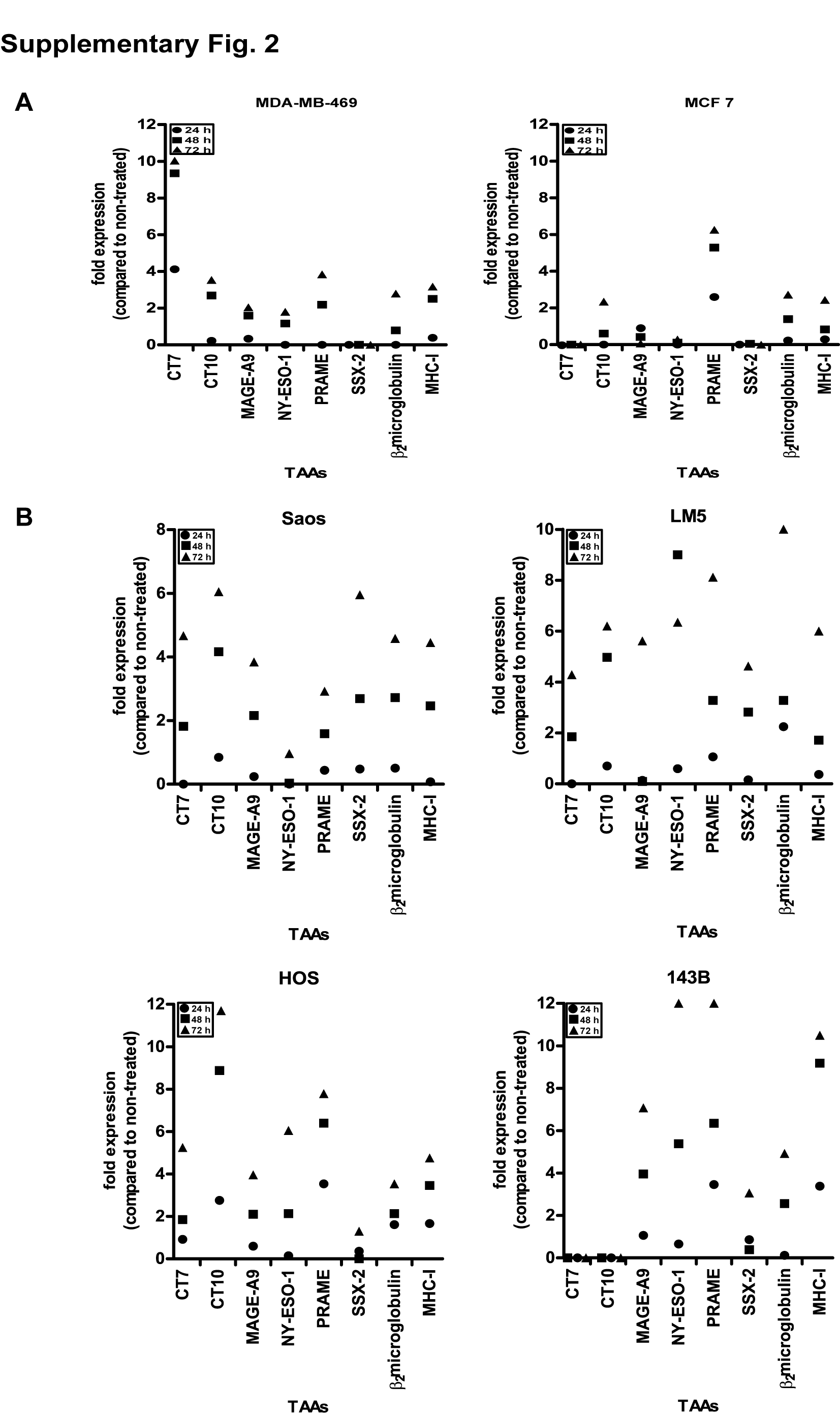

Supplement: Figure S2 — Fractionated γ-radiation results in a time-dependent up-regulation of CT-antigens and MHC-I molecules in vitro . Established cancer cell lines were exposed to fractionated irradiation of 2 Gy on 10 consecutive days to obtain a cumulative dose of 20 Gy. (A) breast cancer cell lines, (B) osteosarcoma cell lines. All Ct values are normalized to 18S rRNA and the data are presented as the fold increase of expression in irradiated (at 24 h, 48 h and 72 h from the last dose of irradiation) compared to the corresponding untreated samples. (TIF) [file pone.0028217.s002.tif]

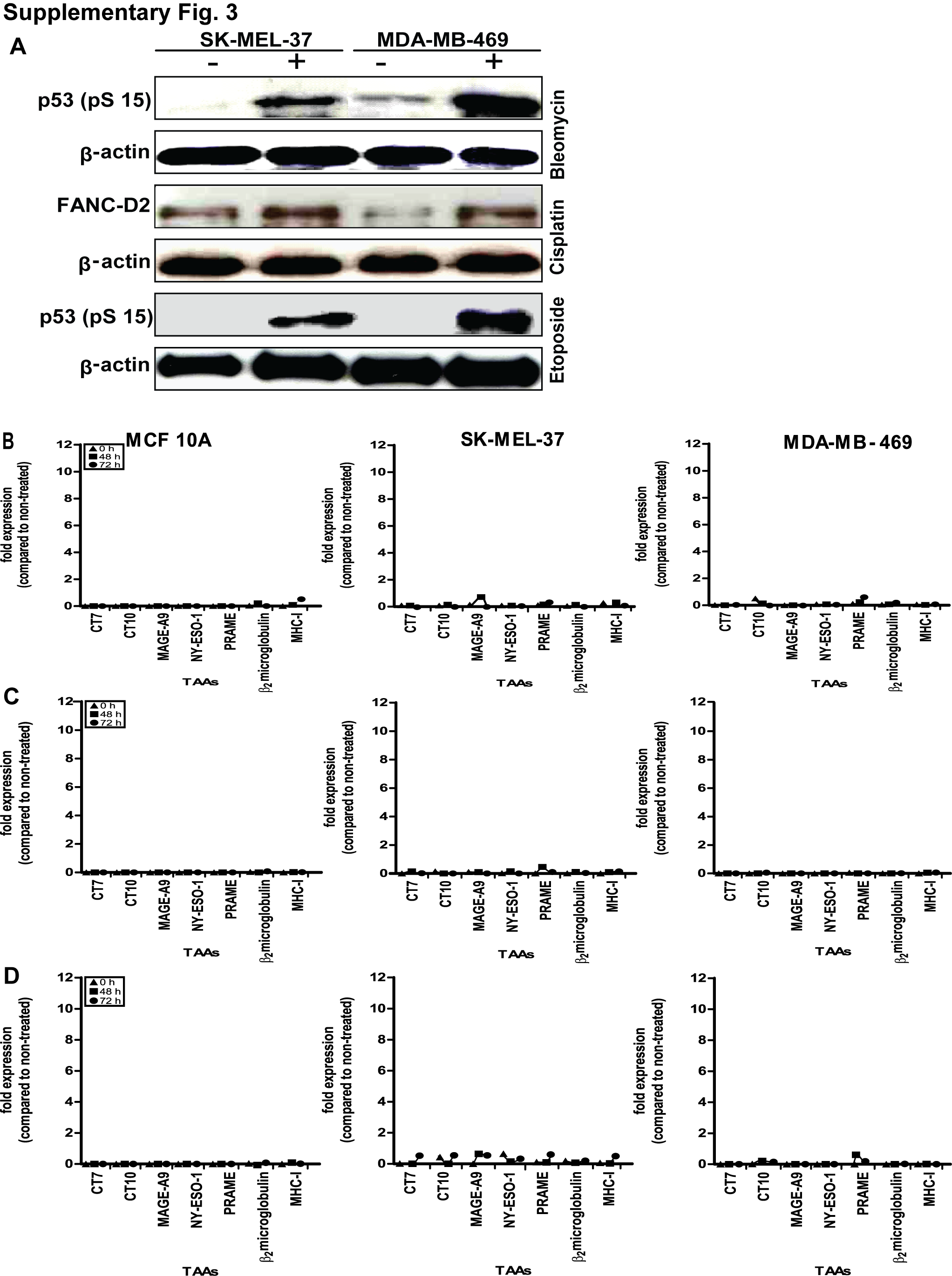

Supplement: Figure S3 — Genotoxic stress has no impact on the expression of CT-antigens or MHC-I in vitro. The breast cancer cell line MDA-MB-469 and the melanoma cell line SK-MEL-37 and the normal breast cell line MCF 10A were exposed to other forms of stress and gene expression was analyzed after 72 h treatment with DNA-damaging agents. (A) Treatment with DNA-damaging agents followed by immunoblotting to detect the activation of hallmark genes p53 and FANC-D2. The same samples were also subjected to RT-qPCR analysis for the expression of CT-antigens and MHC-I at different time points following treatment with (B) bleomycin, (C) cisplatin and (D) etoposide. All Ct values are normalized to 18S rRNA and the data are presented as the fold increase of expression in treated compared to the corresponding untreated samples. (TIF) [file pone.0028217.s003.tif]

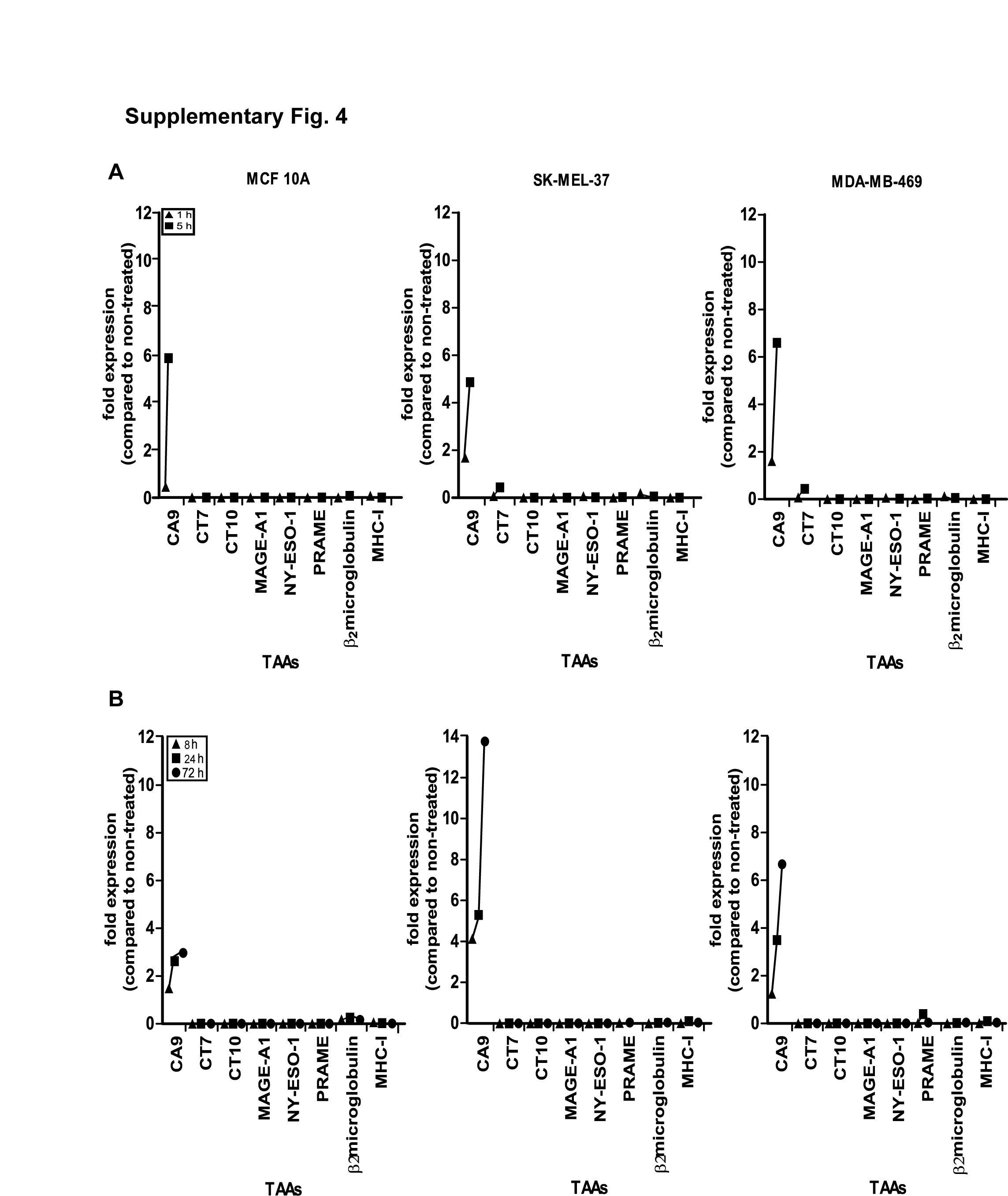

Supplement: Figure S4 — Other forms of stress have no impact on the expression of CT-antigens or MHC-I in vitro. MDA-MB-469 and SK-MEL-37 cells were exposed to (A) hyperthermia and (B) hypoxia and the gene expression following treatment was monitored at different time points by RT-qPCR analysis. All Ct values are normalized to 18S rRNA and the data are presented as the fold increase of expression in treated compared to the corresponding untreated samples. (TIF) [file pone.0028217.s004.tif]

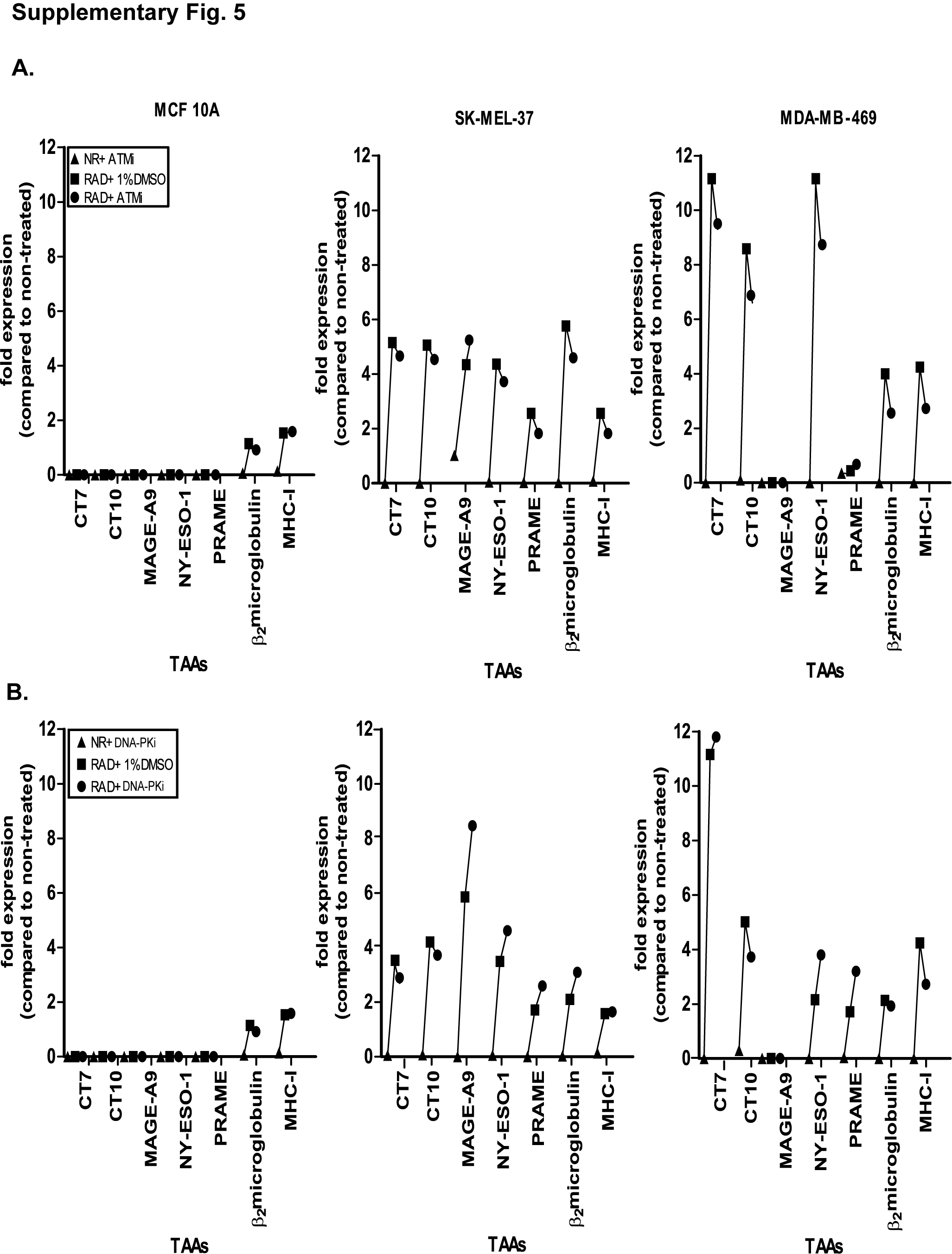

Supplement: Figure S5 — γ-radiation induced expression of CT-antigens and MHC-I is not dependent on the activation of ATM, DNA-PK signaling pathways. The breast cancer cell line MDA-MB-469, the melanoma cell line SK-MEL-37 and the normal cell line MCF 10A were exposed or not to a single dose γ-radiation of 20 Gy in the presence or absence of specific inhibitors of the DNA-damage repair pathways (A) ATM or (B) DNA-PKcs, followed by RT-qPCR analysis for gene expression 72 h following irradiation. All Ct values are normalized to 18S rRNA and the data are presented as the fold increase of expression in treated compared to the corresponding untreated samples. (TIF) [file pone.0028217.s005.tif]
