# Supplementary material for: γ-Radiation Promotes Immunological Recognition of Cancer Cells through Increased Expression of Cancer-Testis Antigens In Vitro and In Vivo
Source: PLoS One. 2011 Nov 29;6(11):e28217. doi: 10.1371/journal.pone.0028217 (PMC3226680; doi:10.1371/journal.pone.0028217)
Supplement: Table S1 — CT-antigens and MHC-I expression in fresh tumor biopsies following ex vivo radiation. Fold increase in expression of CT-antigens and MHC-I in fresh tumor biopsies following 20 Gy ex vivo γ-radiation. All characters in bold represent up-regulation following ex vivo γ-radiation. ND: indicates non-detected. All Ct values are normalized to 18S rRNA and the data are presented as the fold increase of expression in treated compared to the corresponding untreated samples. (DOC) [file pone.0028217.s006.doc]

| **Diagnosis**  **Supplementary Table 1** | **Patient**  **number** | **CT7** | **CT10** | **LAGE-A1** | **MAGE-A9** | **NY-ESO-1** | **PRAME** | **SSX-2** | **β_2_microglobulin** | **MHC-I** |
| --- | --- | --- | --- | --- | --- | --- | --- | --- | --- | --- |
| **Appendix**  **adenocarcinoma** | 1  2  3  4  5  6  7  8  9  10 | ND  **14.21**  ND  ND  ND  0.42  ND  ND  ND  ND | ND  **6.96**  ND  ND  ND  **3.16**  ND  ND  ND  ND | ND  ND  ND  ND  **5.29**  ND  ND  ND  ND  ND | ND  ND  ND  ND  ND  ND  ND  ND  **9.73**  **3.72** | 0.04  **9.12**  0.21  ND  0.32  0.08  0.24  ND  **7.5**  **3.76** | ND  0.04  1.66  ND  ND  **9**  ND  ND  ND  ND | ND  ND  ND  ND  ND  ND  ND  ND  ND  ND | 0.43  **14.44**  0.60  **3.84**  0.81  3.13  0.57  0.03  **3.53**  0.01 | 0.92  **14.59**  0.77  **3.80**  0.49  **2.25**  0.5  0.43  **4.23**  **4.2** |
| **Colon**  **adenocarcinoma** | 11  12 | 0.42  ND | **3.16**  ND | ND  ND | ND  ND | 0.08  0.29 | **9**  ND | ND  ND | **3.13**  **2.95** | **2.25**  **2.9** |
| **Lung**  **adenocarcinoma** | 13  14 | ND  **12.04** | ND  **3.42** | ND  ND | ND  **12.5** | 0.12  **2** | ND  0.65 | ND  0.92 | 0.26  0.53 | 0.01  0.5 |
| **Melanoma** | 15 | **3.2** | 0.06 | ND | **5.52** | **2.16** | 0.32 | ND | 0.03 | 0.73 |
| **Mesothelioma** | 16  17 | ND  ND | ND  ND | ND  ND | **3.27**  ND | **10.75**  **2.31** | ND  ND | ND  ND | **2.40**  0.42 | **5.90**  0.01 |
| **Ovarian adenocarcinoma** | 18  19 | ND  ND | ND  ND | ND  ND | **3.27**  ND | **10.75**  **2.13** | ND  ND | ND  ND | **2.4**  0.42 | **5.9**  0.01 |
| **Pancreatic**  **adenocarcinoma** | 20 | ND | ND | ND | ND | **2.04** | **12.46** | ND | **6.25** | **7.6** |
| **Rectal**  **adenocarcinoma** | 21 | ND | 0.11 | ND | 0.06 | 1.32 | ND | ND | **4.71** | **6.50** |
| **Sarcoma** | 22  23 | ND  ND | ND  ND | 1.61  ND | 1.90  **3.72** | **14.06**  **3.76** | 0.62  ND | ND  ND | 1.46  0.01 | 0.04  **4.2** |

**Scale**

5
