# Supplementary material for: γ-Radiation Promotes Immunological Recognition of Cancer Cells through Increased Expression of Cancer-Testis Antigens In Vitro and In Vivo
Source: PLoS One. 2011 Nov 29;6(11):e28217. doi: 10.1371/journal.pone.0028217 (PMC3226680; doi:10.1371/journal.pone.0028217)
Supplement: Table S2 — Increased expression of CT-antigens and lymphocyte infiltration in sarcoma patients after radiotherapy. (A) Information of sarcoma patients treated with radiotherapy with immunohistochemical scores for T cell infiltration and expression of CT-antigens. All characters in bold represent up-regulation following radiotherapy. MPNST: malignant peripheral nerve sheath tumour, NOS: not otherwise specified. NR indicates non-radiated and RAD indicates corresponding irradiated sections. (DOC) [file pone.0028217.s007.doc]

| **Patient. No**  **Supplementary Table 2**  **A** | **Diagnosis** | **CT7**  NR RAD | **CT10**  NR RAD | **NY-ESO-1**  NR RAD | **CD4**  NR RAD | **CD8**  NR RAD | **Granzyme**  NR RAD |
| --- | --- | --- | --- | --- | --- | --- | --- |
| A | Leiomyosarcoma | **0.5 1** | **0.5 2** | 0 0 | **0 14** | **1 8** | **0 5** |
| B | Leiomyosarcoma | 0 0 | 0 0 | 0 0 | **1 21** | **5 12** | **3 13** |
| C | Leiomyosarcoma | 0 0 | 0 0 | 0 0 | **3 4** | 5 5 | **1 2** |
| D | Myxofibrosarcoma | 0 0 | 0 0 | 0 0 | **1 50** | **2 17** | **1 18** |
| E | Myxoid liposarcoma | **0.5 4** | 0 0 | **3 5** | **0 8** | **0 4** | 1 1 |
| F | Myxoid liposarcoma | 0 3 | 0 0 | 0 0 | **1 2** | 1 1 | 1 1 |
| G | MPNST | 0 0 | 0 0 | 0 0 | 0 0 | 1 1 | **1 2** |
| H | Pleomorphic sarcoma NOS | 0 0 | 0 0 | 0 0 | 1 1 | 6 6 | **1 2** |
| I | Pleomorphic sarcoma NOS | **0 1** | 0 0 | 0 0 | **1 3** | **2 4** | **1 4** |
| J | Pleomorphic sarcoma NOS | **0 0.5** | **0 0.5** | 0 0 | **3 6** | **15 16** | **3 6** |
| K | Pleomorphic sarcoma NOS | 0 0 | 0 0 | **0 3** | **1 7** | **5 16** | **5 24** |
| L | Pleomorphic sarcoma NOS | 0 0 | 0 0 | 0 0 | **4 21** | **1 2** | **0 1** |
| M | Spindle cell sarcoma | 0 0 | 0 0 | 0 0 | **1 42** | **1 22** | **1 5** |
| N | Synovial sarcoma | 0 0 | 0 0 | 0 0 | 1 1 | **1 4** | 1 1 |
| O | Synovial sarcoma | 0 0 | 0 0 | **3 4** | 1 1 | **0 2** | 1 1 |
