# Supplementary material for: γ-Radiation Promotes Immunological Recognition of Cancer Cells through Increased Expression of Cancer-Testis Antigens In Vitro and In Vivo
Source: PLoS One. 2011 Nov 29;6(11):e28217. doi: 10.1371/journal.pone.0028217 (PMC3226680; doi:10.1371/journal.pone.0028217)
Supplement: Table S3 — Radiotherapy up-regulates the expression of MHC-I in sarcoma patients. MHC-I expression in sarcoma patients following radiotherapy. All characters in bold represent up-regulation following radiotherapy. NR indicates non-radiated and RAD indicates corresponding irradiated sections. (DOC) [file pone.0028217.s008.doc]

**Supplementary Table 3**

| **Patient. No** | **MHC-I**  **NR RAD** |
| --- | --- |
| A | **- ++** |
| B | **++ +++** |
| C | + + |
| D | **+ +++** |
| E | **+ ++** |
| F | + + |
| G | **- +** |
| H | ++ ++ |
| I | **+ +++** |
| J | +++ +++ |
| K | **+ +++** |
| L | + + |
| M | **+ ++** |
| N | **+ ++** |
| O | + + |
