# Supplementary material for: γ-Radiation Promotes Immunological Recognition of Cancer Cells through Increased Expression of Cancer-Testis Antigens In Vitro and In Vivo
Source: PLoS One. 2011 Nov 29;6(11):e28217. doi: 10.1371/journal.pone.0028217 (PMC3226680; doi:10.1371/journal.pone.0028217)
Supplement: Table S4 — Quantification of CT-antigen and MHC-I expression in sarcoma patients. (DOC) [file pone.0028217.s009.doc]

**Supplementary Table 4**

| **Scale** | **Intensity**  **of staining** |
| --- | --- |
| - | no expression |
| + | weakly positive |
| ++ | moderately positive |
| +++ | strongly positive |

| **Scale** | **% + ve cells** |
| --- | --- |
| 0 | 0 |
| 0.5 | < 0.1 - 5 |
| 1 | 6 - 10 |
| 2 | 11 - 25 |
| 3 | 26 - 50 |
| 4 | 51 - 75 |
| 5 | < 75 |
